# Influence of ion beam surface treatment on the emission performance of photocathodes

**DOI:** 10.1039/d2na00338d

**Published:** 2022-06-27

**Authors:** Yanwen Liu, Fen Li, Hong Tian, Guojian Wang, Xiaoxia Wang

**Affiliations:** Aerospace Information Research Institute, Chinese Academy of Sciences Beijing 101407 China liuyanwen58@sina.com

## Abstract

Photocathodes are mainly used in such hi-tech fields as photoelectric conversion devices, radiation detection, and accelerators. Laser-driven photocathodes are characterized by low emission, high brightness, easy control, rapid response, *etc.*, and are hopeful to become satisfactory electron sources for next-generation high-frequency miniaturized electric vacuum microwave devices, to effectively improve the performance and rapid response capability of the devices. For this reason, based on previous research efforts on photocathodes, we proposed an idea that ion beam surface treatment technology was used to modify the substrate surface of photocathodes and make the surface textured, enhancing the light absorptivity and alkali metal adsorption performance of photocathodes, so as to improve their emission performance. The surface appearance of photocathodes was analyzed using the scanning electron microscope (SEM) method, and it was found that the surface of oxygen-free copper treated by ion bombardment had a nanocone structure. The photoemission characteristics of photocathodes before and after the treatment of the surface of oxygen-free copper were studied. Before and after the treatment, the maximum photoemission current densities under stable emission performance of the photocathode were 60.5 mA cm^−2^ and 146.0 mA cm^−2^, respectively, and the calculated quantum efficiencies were 2.67 × 10^−3^ and 1.71 × 10^−2^, respectively. The quantum efficiency of the photocathode was increased by 5.41 times after the ion surface treatment. The results show that the photoemission quantum efficiency of the oxygen-free copper surface was increased greatly after modification. It was believed through analysis that the main cause for the increase in quantum efficiency was the enhancement of light absorptivity and the increase in the emission surface area.

## Abstract 2

Photocathodes are mainly used in such hi-tech fields as photoelectric conversion devices, radiation detection, and accelerators.

## Graphical abstract

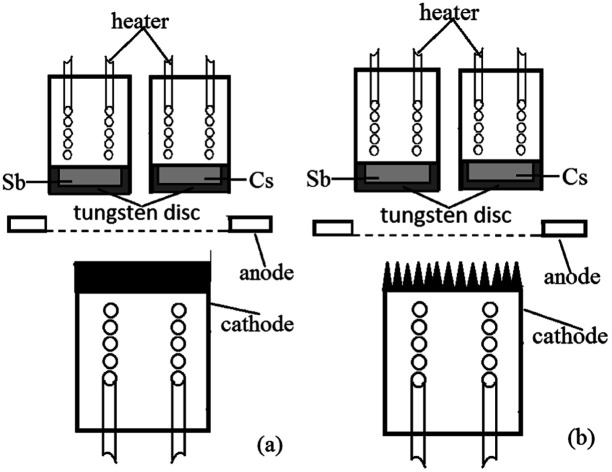

## 1 Introduction

Vacuum microwave devices are widely used in such aspects as satellite communication and particle accelerators.^1^ New requirements for such operating characteristics as power, frequency, and bandwidth have been put forward in modern microwave technology,^2,3^ so research on novel electron emitters meeting microwave source requirements is of important significance for the development of such technologies as satellite communication and microwave devices.^4^

At present, the electron emitters used in electric vacuum microwave devices are dominated by thermionic cathodes, including scandate cathodes,^5,6^ oxide cathodes,^7,8^ and film-coated cathodes.^9^ M-type cathodes are electron emitters mostly studied and applied at present.^10,11^ However, an M-type cathode has relatively high operating temperature and a relatively large evaporation rate, so field emission cathodes have attracted the attention of researchers.^12,13^ Carbon nanotube field emission cathodes have been used in travelling wave tubes,^14^ but such cathodes have uneven emission and poor stability; therefore, we propose to study photocathodes used for microwave devices.^15^

An important application of photocathodes is photoelectric conversion devices, such as photoelectric tubes, image intensifiers, and camera tubes. The discovery of multialkali photocathodes makes photocathodes more widely used in such hi-tech fields as radiation detection and photography.^16^ The emergence of photocathode injectors makes photocathodes applied in such aspects as accelerators.^17^ At present, pulsed laser-driven photocathodes mainly have such types as metal, metal oxide, alkali metal semiconductor, and GaAs negative electron affinity.^18,19^ In recent years, the excellent intrinsic attributes (wide band gap, direct transition, and strong chemical stability) of the GaN photoelectric material make it a satisfactory ultraviolet photocathode material, attracting great attention of people.^20,21^

Laser-driven photocathodes have many features, such as low emittance, high brightness, easy control, and rapid response, and they are hopeful to become satisfactory electron sources for next-generation high-frequency miniaturized electric vacuum microwave devices, effectively raising the response speed of these devices and thus improving the performance and rapid response capability of the devices. For this reason, based on previous research efforts on photocathodes, we proposed an idea that the ion beam surface treatment technology was used to modify the substrate surface of photocathodes, enhancing the adsorption performance and light absorptivity of photocathodes, so as to increase their emission current density.

The experiment conducted by the NASA Lewis Research Center and some other experiments demonstrated that the material surface undergoing ion beam treatment would have burrs on the nanometer scale, to reduce the secondary electron emission coefficient of the material.^22,23^ These burrs on the nanometer scale were very small in size, equivalent to the de Broglie wavelength of electrons. The decrease in the scale made the macroscopically fixed quasi-continuous energy band disappear, with discrete energy levels exhibited, so the quantum size effect was quite evident, causing the nano-material to have physical and chemical properties different from those of block materials, such as surface effect, and small size effect.^24,25^ Study on the emission characteristic of nano-particle films has become a hot spot in the international research field.^26,27^ We studied the thermionic emission uniformity^28^ and thermal radiation characteristics^29^ of a nano-particle film and found that such a material had quite good emission uniformity and very strong thermal radiation characteristics. At present, there are no reports on the study on increasing the emission current density of photocathodes through ion beam surface treatment. In this paper, we studied the application of ion beam surface modification technology in photocathodes using the basic theory of photoemission, and this is of quite important academic value and application value for the theory and application of photoelectron emission and the expansion of application and study fields of ion beam surface modification technology.

## 2 Preparation of the photocathode

### 2.1 Structure of the photocathode

Photoelectrons can be generated by a laser-excited cesium antimonide (Cs_3_Sb) photocathode with a low photon energy and visible light wavelength, and such a photocathode has high quantum efficiency and its preparation process is relatively simple, so we selected a Cs_3_Sb photocathode for modification study. Alkali metal antimonide photocathodes generally have two categories based on the structure, that is, cubic structure and hexagonal structure. The former category has good photoemission characteristics, and the latter one has poor photoemission characteristics. Since a cubic crystal has a larger atom density than a hexagonal crystal, the crystal with a large atom density has a large electron concentration, and both light absorption and photoelectric excitation are proportional to electron concentration, so all current practical alkali metal antimonide photocathodes have a cubic crystal structure.^30^ The Cs_3_Sb photocathode material studied in this paper has a body-centred cubic structure, with a lattice constant of 0.9128 nm.^31^ The Cs_3_Sb photocathode is a well-defined material in crystallography with a cesium-antimony atom ratio of 3 : 1. For a traditional reflective Cs_3_Sb photocathode, an antimony film is deposited on its substrate surface first, and oxygen-free copper is generally selected for the substrate of the photocathode. And then, an alkali metal source is heated, and the alkali metal vapor on the photocathode surface acts on the antimony film.

**Fig. 1:**
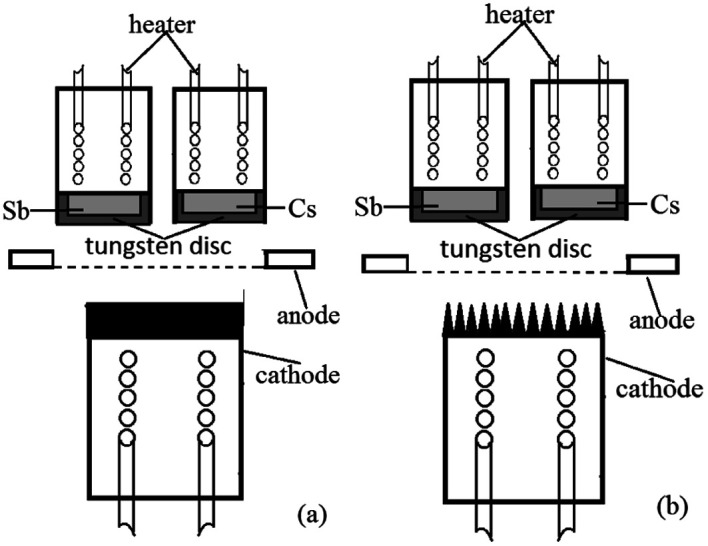
Structural schematic diagram of the reflective Cs_3_Sb photocathode: (a) untreated oxygen-free copper substrate and (b) ion-textured oxygen-free copper substrate.

For a Cs_3_Sb photocathode, the accelerated residual gas ions or laser will make the alkali metal in the photocathode desorbed, causing the stability of the photocathode to worsen and the life to shorten. To solve these problems and to meet the requirements for photocathodes with large current density, we proposed an idea that ion beam surface treatment technology was used to modify the substrate surface of the photocathode, enhancing the light absorptivity and alkali metal adsorption performance of the photocathode. The structure of the reflective Cs_3_Sb photocathode studied in this paper is shown in Fig. 1.

The photocathode comprised a heater for heat activation, alkali metal cesium source, antimony source, photocathode substrate, anode, *etc.* The cesium source was a mixed powder of cesium salt (Cs_2_CrO_4_) and a reducing agent, and the antimony source was antimony powder with a purity of 99.999%. The anode had a mesh grid structure. The photocathode substrate was made into a cylinder with oxygen-free copper, and its outer bottom surface underwent ion beam surface modification.

### 2.2 Ion beam surface modification

The sputtering rate of ions has a lot to do with the sputtering coefficient of the material. When ions with some energy bombard a material surface with a high sputtering coefficient, and there is a certain amount of another material with a low sputtering coefficient deposited on the surface, and it is possible to form a highly textured surface, which has burrs with a certain interval and height. When an oxygen-free copper surface undergoes ion bombardment, a certain number of molybdenum atoms deposited will form such a texture. Since the sputtering coefficient of molybdenum atoms is lower than that of oxygen-free copper, there are tiny regions with molybdenum atoms aggregated on the oxygen-free copper surface. These tiny regions act as masks, and the copper atoms around the regions are rapidly sputtered by ions, and thus a burr structure is formed.

**Fig. 2:**
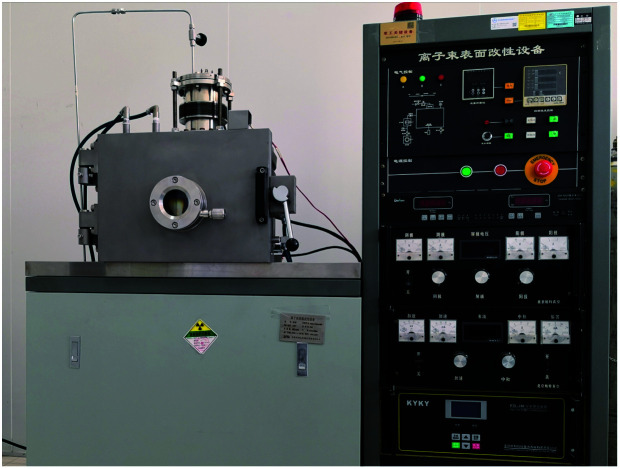
Picture of the ion beam surface modification apparatus.

Through process experiments, the distance between the molybdenum target and sample was adjusted, and the heater, sample table, molybdenum target and mold were improved, to develop an ion beam surface modification apparatus for the oxygen-free substrate of the photocathode, as shown in Fig. 2.

The main purpose of the ion beam surface modification apparatus was to provide a sputtering ion beam with a given beam current density and energy, and to deposit molybdenum atoms with a given rate. We selected a Kaufman ion source with a beam energy of 2.0 keV and a diameter of 6 cm, which simultaneously had sputtering and deposition functions. The ion source was on the top of the vacuum chamber, and the annular molybdenum target was placed above the sample table. The designed sample table had planetary rotation function and could be heated to 600 °C. To control and adjust the deposition rate of molybdenum atoms, the molybdenum target was designed into a toothed structure and had an angle with the horizontal plane. The deposition rate of molybdenum atoms could be adjusted by adjusting the total area ratio of tooth wing to ring.

**Fig. 3:**
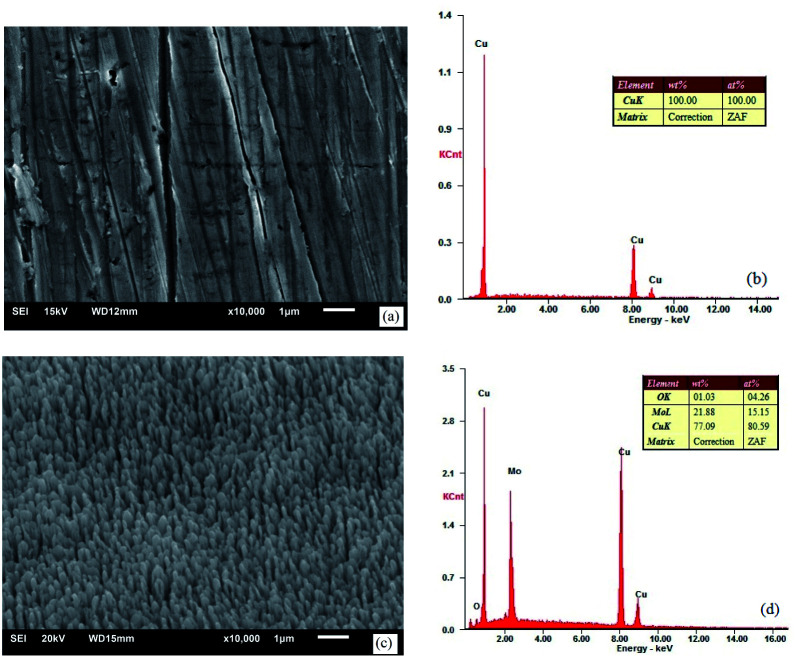
SEM images and EDS spectra: (a) and (b) untreated oxygen-free copper surface and (c) and (d) ion-textured oxygen-free copper surface.

When the vacuum reached 5 × 10^−4^ Pa, a gas not reacting with the metal, *e.g.*, argon, was filled into the system, to make the pressure 0.01–1 Pa. The size of burrs could be changed by adjusting the sputtering rate and gas filling pressure. Fig. 3 shows the SEM images and components of the oxygen-free copper surface before and after treatment.

**Fig. 4:**
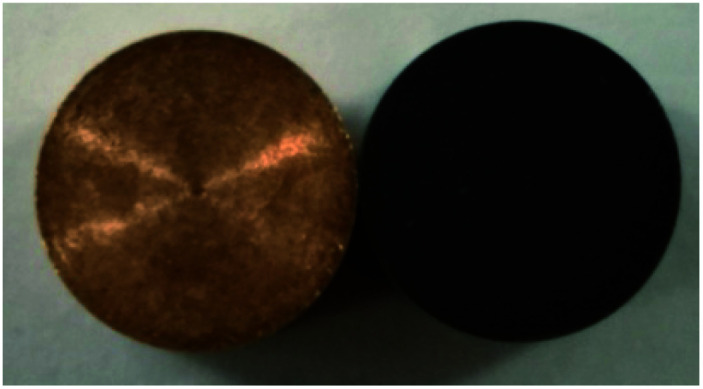
Picture of untreated and treated oxygen-free copper photocathode substrate surfaces.

Fig. 3(a) and (b) show that, except some lathing lines, the untreated oxygen-free copper surface was relatively flat and smooth without impurities. Fig. 3(c) and (d) show the oxygen-free copper surface after ion beam surface modification under the following conditions: pressure of 0.02 Pa, heating temperature of 400 °C, voltage of 1.4 kV and sputtering target material of molybdenum. The surface exhibited texture appearance and formed a dense nanocone array, with burrs having a diameter of ∼150 nm, characteristic height of ∼400 nm and interval of ∼200 nm. In addition to copper, there were also sputtered molybdenum and adsorbed oxygen on the surface. Fig. 4 shows a picture for comparison between untreated and treated oxygen-free copper photocathode substrate surfaces.

As can be seen from Fig. 4, the treated oxygen-free copper photocathode substrate surface is much darker than the untreated oxygen-free copper photocathode substrate surface and exhibits a black color. The reason is that the nanocone burr texture has very strong light absorption capability. Therefore, the ion beam bombardment technology was used to modify the substrate surface of the photocathode, enhancing the light absorptivity of the photocathode and thus to improve its photoemission performance.

### 2.3 Preparation of the photocathode

**Fig. 5:**
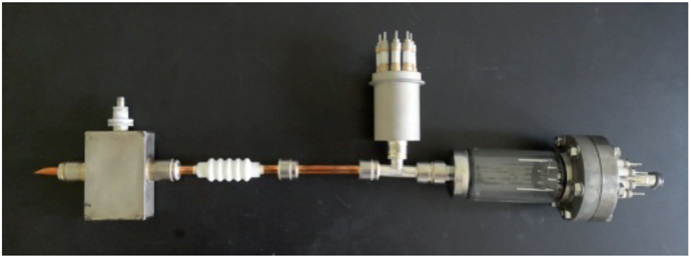
Photocathode test device.

The substrate of the photocathode, antimony source, cesium source, *etc.* were welded to the electrode flange of the test device, and the structure is shown in Fig. 5.

The photocathode test device comprised a light input window, thermionic cathode ionization gauge, photocathode, electrode, anode, ion sputtering pump, and exhaust port. The ionization gauge should undergo electron bombardment degassing in a vacuum, and it could measure the vacuum degree in a sealed device.^32^ Both the cathode and anode were fixed to one flange, so as to conveniently control the distance and parallelism between them. The cathode had a diameter of 5.9 mm, and the molybdenum sheet anode had a round hole with a diameter of 5 mm in the center, in which a mesh grid was welded and of which the light transmittance could reach over 95%. The distance between the cathode and anode was 1 mm to reduce the influence of cathode edge emission on the cathode emission.

**Fig. 6:**
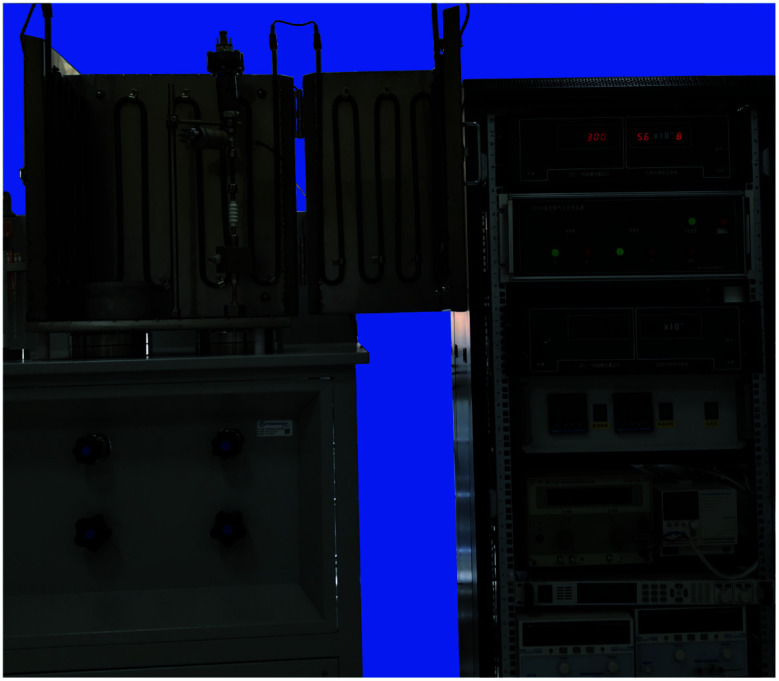
Photocathode preparation device.

The photocathode preparation device was connected to the exhaust equipment, and the exhaust vacuum system comprised an ion pump, molecule pump, *etc.* The device is shown in Fig. 6.

After the photocathode preparation device underwent 40 hours of degassing at 450 °C, its limit vacuum degree could reach 4.8 × 10^−8^ Pa. After sealing, the small titanium ion pump for suction and the vacuum gauge were turned on, and the vacuum degree could be 6 × 10^−8^ Pa, which met the requirement for the vacuum degree during the operation of the photocathode preparation device. At this moment, photocathode preparation could be carried out.

Firstly, the oxygen-free copper substrate of the photocathode was heated to 600 °C, which was maintained for one hour to evaporate the gas impurities adsorbed on the substrate surface of the photocathode, and the temperature was lowered to ∼130–150 °C. Then, the antimony source was heated to evaporate the antimony on the substrate surface of the photocathode, which formed a film. Finally, the cesium source was heated to evaporate cesium into vapor, which flowed to the surface of the photocathode and reacted with the antimony film. The photocurrent was increased slowly to reach its peak in half an hour to one hour by monitoring the white light photocurrent, adjusting the temperature of the cesium source and controlling the evaporation rate of cesium stream.

## 3 Test results of the emission performance of the photocathode and discussion thereof

The photoemission performance of the photocathode was tested with a photocathode emission test system. In the experiment, a semiconductor laser was used, because it was characterized by low power consumption, long life, reliable performance, high output laser beam quality, *etc.*, and the laser was a continuous wave with a wavelength of 532 nm and beam diameter of 1 mm.

The laser was turned on, and the laser irradiated the surface of the cathode through the light input window and the anode mesh grid. High voltage was applied slowly between the cathode and anode and it finally reached 500 V. The pressure in the system was of the order of 10^−7^ Pa in the experiment.

**Fig. 7:**
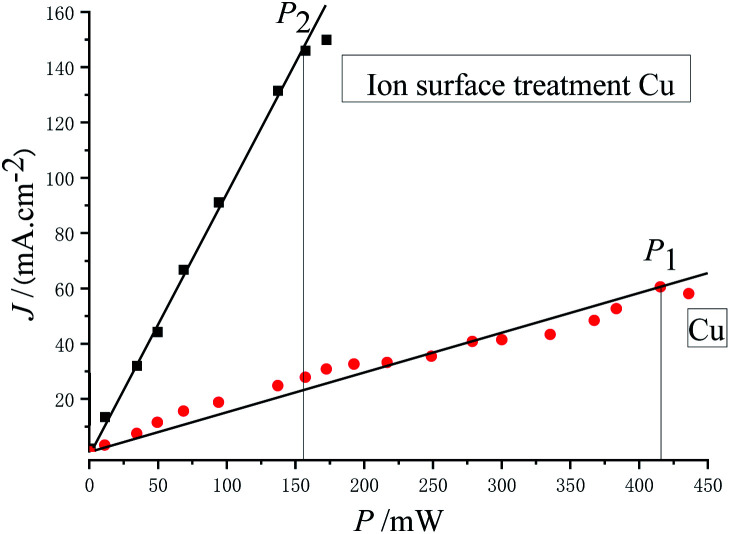
Photoemission current density *versus* laser power.

Fig. 7 shows the relationship between photoemission current density and laser power.

As can be discerned from Fig. 7, the photoemission current density is proportional to the laser power. The maximum laser powers with stable emission obtained before and after substrate treatment were *P*_1_ = 416 mW and *P*_2_ = 157 mW, respectively, and the following was obtained: *P*_1_/*P*_2_ = 2.65.

The main factors influencing the stability of the cesium antimonide photocathode are temperature and the vacuum degree. The cesium in the photocathode material will be separated or desorbed with the increase in temperature, and the residual gas in the vacuum chamber will also be toxic to the photocathode material. During test in this experiment, the vacuum degree of the device was ∼1 × 10^−7^ Pa, so the residual gas had a very small influence on the attenuation of emission current. Therefore, the main factor causing the attenuation of emission current of the photocathode in this experiment should be the temperature rise caused by the absorption of laser energy. Thus it can be believed that the light absorptivity of the photocathode after ion beam surface modification is increased by 1.65 times, compared with the unmodified photocathode.

The emission capability of the photocathode material is normally described with quantum efficiency *Y*:1
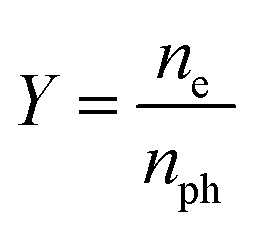
where *n*_e_ is the number of photoelectrons:2
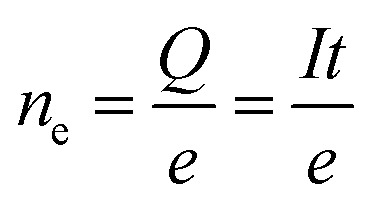
*n*_ph_ is the number of photons:3
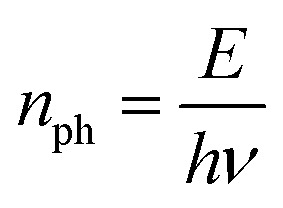


If the wavelength of the laser is 532 nm, then *hν* = 2.34 eV; therefore4
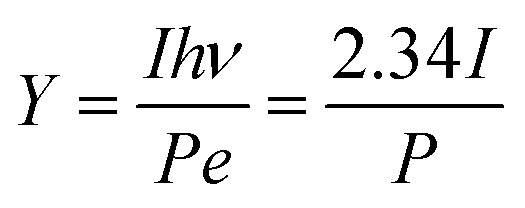


In this experiment, the maximum laser powers with stable emission of the photocathode obtained before and after substrate treatment were *P*_1_ = 416 mW and *P*_2_ = 157 mW, respectively, and the photoemission current densities were *J*_1_ = 60.5 mA cm^−2^ and *J*_2_ = 146.0 mA cm^−2^, respectively. The light spot diameter was *Ø* = 1 mm, and the quantum efficiencies calculated with eqn (4) were *Y*_1_ = 2.67 × 10^−3^ and *Y*_2_ = 1.71 × 10^−2^, respectively, that is, *Y*_2_/*Y*_1_ = 6.41, indicating that the quantum efficiency was increased by 5.41 times.

**Fig. 8:**
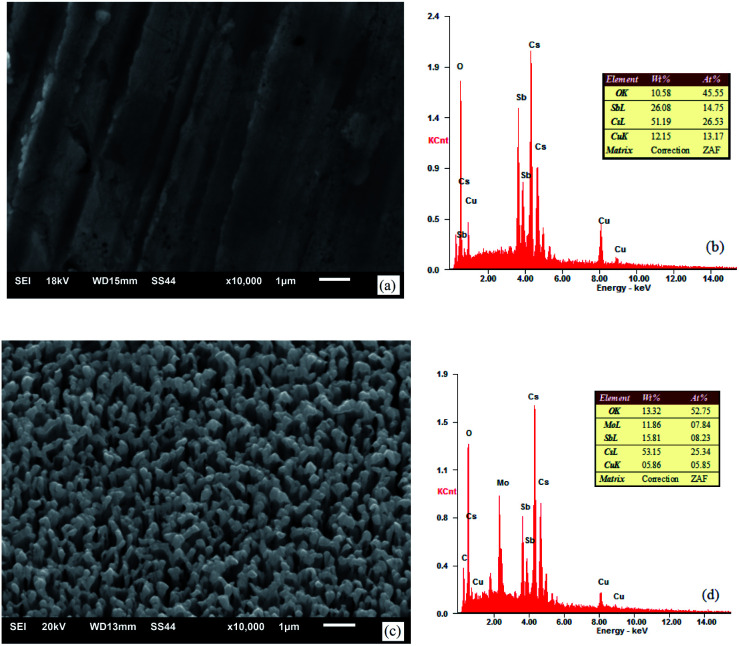
SEM images and EDS spectra of the photocathode: (a) and (b) untreated oxygen-free copper surface and (c) and (d) ion-textured oxygen-free copper surface.

The appearance and components of the surface after the activation of the photocathode are shown in Fig. 8.

As can be seen from the component spectra in Fig. 8, the main components in the surface of the photocathode are Cu, Cs, Sb, Mo, and O adsorbed from air. Before substrate treatment, the percentages of Cs and Sb atoms in the surface of the photocathode were 26.53% and 14.75%, respectively; in other words, the Cs to Sb atom ratio was 1.80. After substrate treatment, the percentages of Cs and Sb atoms in the surface of the photocathode were 25.34% and 8.23%, respectively; in other words, the Cs to Sb atom ratio was 3.08. This indicates that the treated oxygen-free copper surface has stronger Cs adsorbing capability than the untreated one. The reason is that the material in the nanometer burr form has a larger specific surface area and insufficient coordination of surface atoms, which offer stronger adsorption performance, compared with the same material in the block form. Therefore, the treated oxygen-free copper photocathode substrate surface has relatively strong Cs adsorption capability, reducing the desorption of Cs. As a semiconductor cesium antimonide photocathode, when the Cs to Sb ratio is approximately 3–4, the photocathode has a relatively high quantum efficiency.

**Fig. 9:**
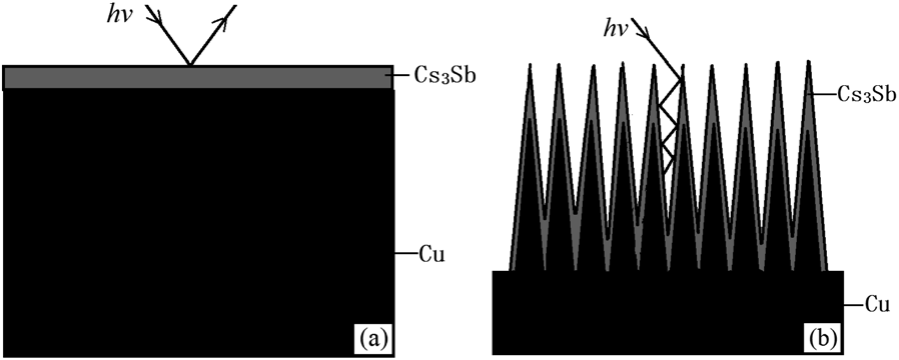
Schematic diagrams of the photocathode surface structure: (a) untreated oxygen-free copper and (b) ion-textured oxygen-free copper.

It can be seen from the SEM images that the untreated photocathode surface is relatively smooth and the photocathode surface undergoing ion beam surface modification undulates on a scale of ∼200 nm. Based on this, the photocathode surface models with different structures can be established, as shown in Fig. 9.

As can be seen from Fig. 9, when photons hit the surface of untreated oxygen-free copper, some of the photons will be reflected back into the vacuum chamber. However, most of the photons emitted to the surface of treated oxygen-free copper hit the sides or bottoms of “burrs”, and are hard to return to the vacuum chamber after several times of reflection, so as to reduce the reflection coefficient of photons, which can be demonstrated by the relatively black color of the treated oxygen-free copper.

**Fig. 10:**
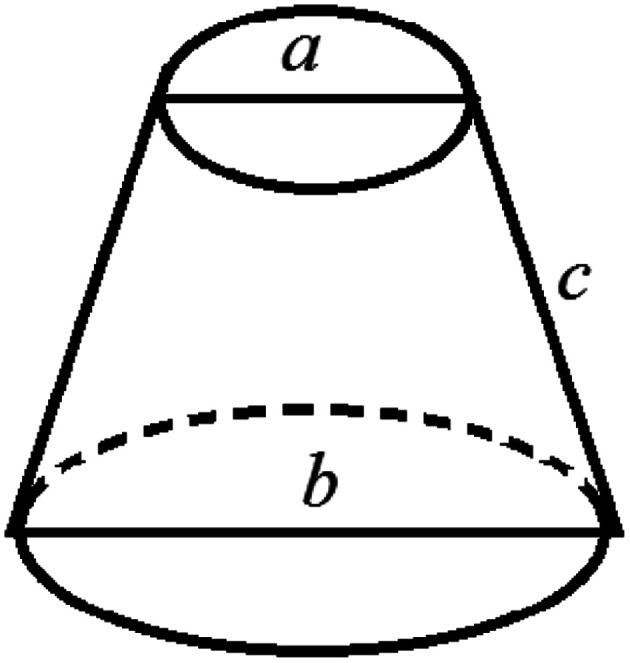
Schematic diagram of the burr model on the oxygen-free copper surface of the photocathode undergoing ion beam treatment.

It is assumed that a protuberance on the surface of the photocathode undergoing ion beam modification has a shape of a circular truncated cone as shown in Fig. 10.

The surface area (*S*_1_) of the circular truncated cone is:5
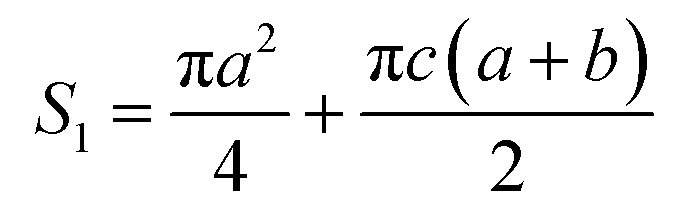


The bottom area (*S*_2_) of the circular truncated cone is:6
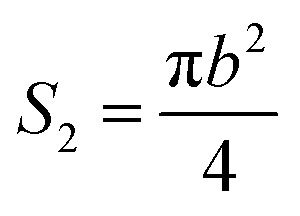


Thus, the ratio of the surface area of the circular truncated cone to the bottom area of the circular truncated cone is:7
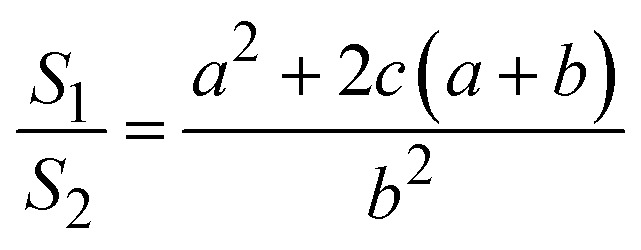


It is very easy to make the side length of the circular truncated cone larger than the bottom diameter of the circular truncated cone, that is, *c* > *b*, by controlling the ion bombardment time, and thus the following can be obtained according to eqn (7):8
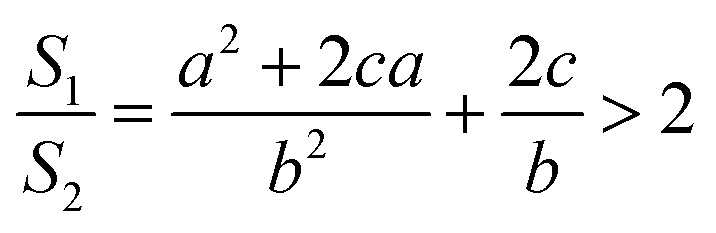


If *a* = 0, in other words, the protuberance is a cone, and then the following is obtained according to eqn (7):9
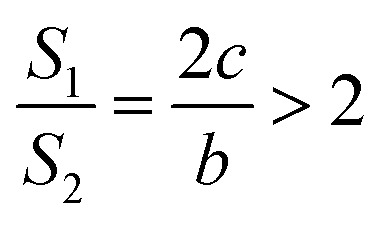


If *a* = *b*, in other words, the protuberance is a cylinder, and then the following is obtained according to eqn (7):10
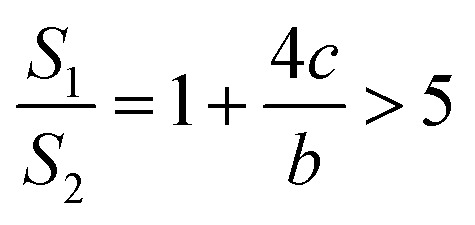


As can be discerned from the above analysis, each protuberance on the surface of the photocathode after ion beam surface modification has a relatively large surface area. This demonstrates that one main cause for the increase in the emission current density of the photocathode after ion beam surface modification is the increase in the surface area, which increases the photoemission current.

The other cause for the improvement of photoemission performance after modification is that when photons hit the sides or bottoms of burrs, most of the reflected photons are captured by the nearby burrs, leading to an increase in light absorptivity.

The quantum efficiency of the modified photocathode is hopeful to be raised further by adjusting the ion bombardment surface modification process and the Sb–Cs ratio. A kind of Na_2_KSb photocathode with better thermal stability can be prepared, and thus the emission current density can be raised further on such a novel photocathode.

## 4 Conclusions

To meet the needs of a high current photocathode for high-frequency miniaturized microwave vacuum devices, a preparation method for novel reflective photocathodes was proposed. Ion bombardment technology was used for surface modification of an oxygen-free copper material commonly used for photocathode substrates, so that a dense nanocone array was formed in the copper material surface, improving the adsorption performance and light absorptivity and increasing the emission area of the photocathode, and thus greatly increasing the photoemission current relative to the untreated photocathode.

The treated oxygen-free copper substrate surface of the photocathode is much darker than the untreated surface and exhibits a black color. This is caused by the very strong light absorption capability of the nanometer burr texture. The surface structure was analyzed using the SEM method. The treated oxygen-free copper surface formed a dense nanocone array with burrs having a diameter of ∼150 nm, height of ∼400 nm and relatively small interval, and the surface had a particle-free, firm and uniform all-metal structure. Accordingly, photocathode surface models with different structures were established.

The photoemission characteristics of the photocathode before and after surface treatment were studied. Before and after the treatment, the maximum photoemission current densities under stable emission performance of the photocathode obtained in the experiment were 60.5 mA cm^−2^ and 146.0 mA cm^−2^, respectively, and the calculated quantum efficiencies were 2.67 × 10^−3^ and 1.71 × 10^−2^, respectively. The quantum efficiency was increased by 5.41 times after surface treatment. The results show that the photoemission quantum efficiency of the oxygen-free copper surface was increased greatly after modification, and there is room for further development of the oxygen-free copper surface. The electron emission mechanisms of different surface structures were analyzed. The main cause for the increase in the quantum efficiency of photocathodes after surface modification was the enhancement of light absorptivity and the increase in the emission surface area. These research results can provide a reference for the development of photocathodes with large current density.

## Author contributions

Y. W. Liu conceived the idea for the research, designed the experiments, performed the growth, and morphological analysis, and wrote most of the manuscript. F. Li developed the mask preparation procedure and provided patterned substrates for the growth. H. Tian performed TEM sample preparation and related structural and compositional analyses and wrote the corresponding section of the manuscript. G. J. Wang significantly participated in the growth, analysis, some device processing and measurements, and wrote the corresponding part of the manuscript. X. X. Wang supervised the work and provided extensive comments on the manuscript. All authors commented on the work and provided valuable input throughout the project and approved the final version of the manuscript.

## Conflicts of interest

There are no conflicts of interest to declare.

## Supplementary Material
